# Study on the treatment of pancreatic cancer with integrated traditional Chinese and Western medicine: A study protocol of a multicenter prospective cohort study

**DOI:** 10.1097/MD.0000000000017975

**Published:** 2019-11-22

**Authors:** Juling Jiang, Rui Liu, Zhenhua Zhang, Xiwen Zhang, Runzhi Qi, Shuntai Chen, Xing Zhang, Yupeng Xi, Qiujun Guo, Honggang Zheng, Baojin Hua

**Affiliations:** a Guang’anmen Hospital, China Academy of Chinese Medical Sciences; b China Academy of Chinese Medical Sciences; c Beijing University of Chinese Medicine, Beijing, China.

**Keywords:** clinical curative effect, clinical plan, integrated traditional Chinese and Western medicine, overall survival, pancreatic cancer

## Abstract

**Introduction:**

Pancreatic cancer is one of the most lethal malignancies worldwide. Most patients are diagnosed at an advanced stage, which leads to a poor prognosis and a low survival rate. At present, treatment options for pancreatic cancer are limited, so it is vital to explore new treatments and strategies. Traditional Chinese medicine (TCM) is an important method for cancer prevention and treatment in China. We will conduct a multicenter, prospective cohort study to evaluate the survival and quality of life of patients with advanced pancreatic cancer treated with integrated traditional Chinese and Western medicine, further refine the core pathogenesis of TCM for pancreatic cancer, form a core prescription, and provide clinical data support for the clinical plan of integrated treatment of pancreatic cancer using Chinese and Western medicine; this will aid in the development of the best comprehensive treatment plan for patients.

**Methods and analysis:**

This study will recruit patients with stage 3 to 4 pancreatic cancer in 12 medical units from April 2019 to June 2020. Patients will be divided into a Western medicine treatment group and an integrated traditional Chinese and Western medicine treatment group, with a total of 148 patients. Overall survival is the main efficacy index, while the secondary efficacy indexes are progression-free survival, tumor markers, TCM symptom grading scale, quality of life assessment, Eastern Cooperative Oncology Group (ECOG) score, and imaging assessment. A follow-up will be performed every 6 weeks ±1 week. The end point is the death of the patient or the end of the study (October 31, 2021). Statistical analysis will be performed using Statistical Packages of Social Sciences software (SPSS).

**Ethics and dissemination:**

This work was supported by Beijing Municipal Science and Technology Commission and approved by the ethics committee of Guang’anmen Hospital, China Academy of Chinese Medical Sciences (Approval No. 2019-016-KY). All patients will sign a written informed consent prior to data collection. The results will be disseminated through peer-reviewed journals and conference presentations and will be openly shared after completion of the trial.

**Trial Registration:**

The trial was registered with the Chinese Clinical Trials Registry (ChiCTR1900022632, pre-registration).

## 1 Introduction

Pancreatic cancer is one of the most lethal malignancies worldwide. According to a GLOBOCAN report, there are an estimated 458,918 new cases, and there were 432,242 cancer deaths in 2018. Of those cases, one-fifth occurred in China.^[1]^ According to the China cancer registry data of 2014, it is the seventh leading cause of cancer death in either sex, with 92,000 new cases and 81,100 deaths each year.^[2]^ The mortality rate is closely related to the incidence rate and is increasing year by year.

So far, although some risk factors have been identified related to the occurrence of pancreatic cancer, such as smoking, diabetes, dietary factors, obesity, alcoholism, age, ethnicity, family history and genetic factors, *Helicobacter pylori* infection, and chronic pancreatitis.^[3]^ But the causes of pancreatic carcinoma are still not fully known.^[4]^ Pancreatic ductal adenocarcinoma (PDAC) is the most common type of pancreatic cancer, and it accounts for approximately 85% of pancreatic malignancies.^[5]^ Pancreatic cancer is insidious with atypical early symptoms. It often manifests as upper abdominal discomfort, lower back pain, indigestion, or diarrhea, and is easily confused with other digestive diseases. There are currently no standard procedures for screening patients at high risk for pancreatic cancer, and most patients have no symptoms before the disease progresses to an advanced stage. Most patients are in the advanced stage at the time of diagnosis, with a natural course of 4 to 6 months, which leads to a poor prognosis and a low survival rate. The global surveillance of trends in cancer survival during 2000 to 2014 shows that age-standardized 5-year net survival estimates were generally in the range of 5% to 15%, and in most countries it is no >15%. In fact, even France, Japan, New Zealand, and many other developed countries are below 10%.^[6]^

Pancreatic cancer treatment options are limited at present; surgery is still the first-line treatment for early pancreatic cancer and is considered to have the potential to result in a cure. Postoperative adjuvant chemotherapy, radiotherapy, and traditional Chinese medicine (TCM) treatment can significantly prolong the survival of patients,^[7–9]^ but only about 20% of patients have the opportunity to have an initial resection.^[10]^ The 5-year survival rate of patients with a complete resection is only 25%, and recurrence and metastasis remain the leading cause of death in most patients.^[11]^ The potential benefits of radiotherapy for locally advanced pancreatic cancer have been extensively studied, and palliative surgery is feasible for patients with locally advanced and metastatic pancreatic cancer but chemotherapy is still the main treatment. However, the efficacy of chemoradiotherapy is limited,^[12]^ so it is urgent to explore new treatment methods or treatment options.

Studies have shown that the median survival of patients with stage III/IV pancreatic cancer treated with Western medicine is 6 to 11 months,^[13,14]^ and the 1-year survival rate is only about 20%. TCM is an important method for cancer prevention and treatment in China. The clinical observation of 59 patients with advanced pancreatic cancer showed that intervention with TCM could prolong the median survival of stage III/IV pancreatic cancer for >2 months.^[9]^ Therefore, it is urgent to accelerate the formation of a clinical plan for the treatment of pancreatic cancer with integrated traditional Chinese and Western medicine.

## 2 Objective and purpose

This study will conduct a multicenter, prospective cohort study to evaluate the survival and quality of life of patients with advanced pancreatic cancer treated with integrated traditional Chinese and Western medicine, further refine the core pathogenesis of TCM for pancreatic cancer, form a core prescription, and provide clinical data support for a treatment plan that integrates Chinese and Western medicine and enables the development of the best comprehensive plan for patients.

## 3 Methods and analysis

### 3.1 Study setting and design

**Figure 1:**
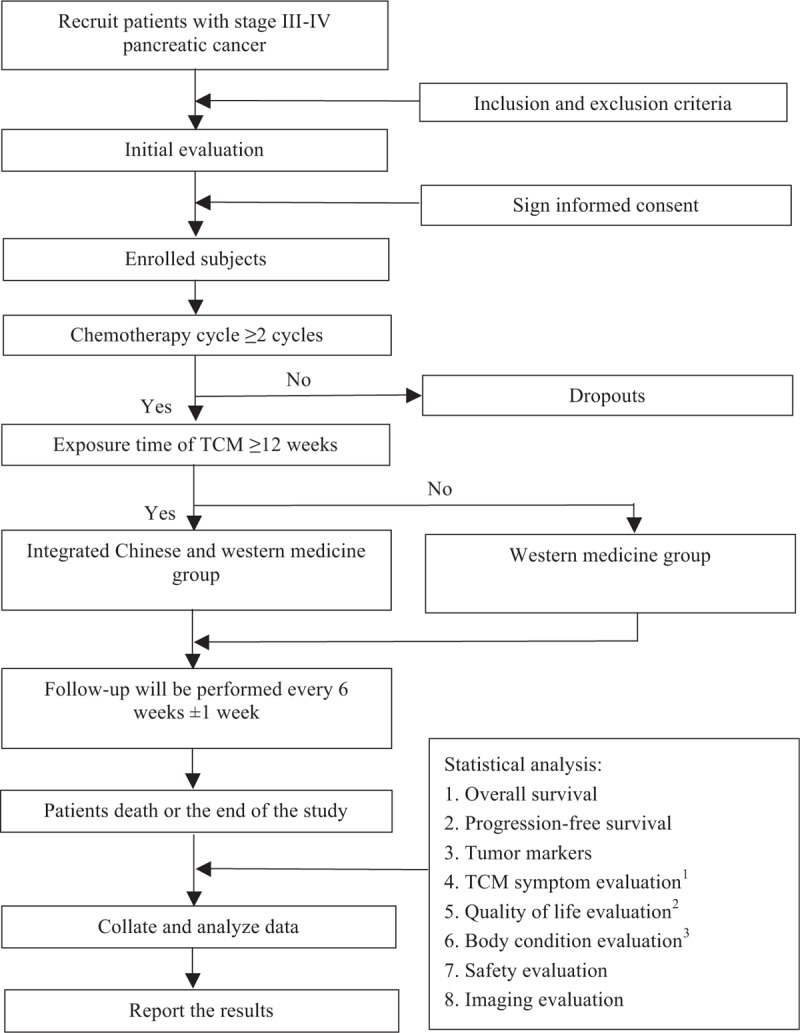
Study schema and flow chart.

This study is a multicenter, prospective cohort study on the clinical efficacy of integrated traditional Chinese and Western medicine in the treatment of pancreatic cancer. It was launched in April 2019 and is scheduled to end in October 2021 with enrollment to be completed by June 31, 2020. All cases will be collected at 12 medical units including Guang’anmen Hospital, China Academy of Chinese Medical Sciences, Henan Provincial Cancer Hospital, Jining No.1 People's Hospital, Jiangsu Provincial Hospital of Chinese Medicine, Zhejiang Provincial Hospital of TCM, Yueyang Hospital of Integrated Traditional Chinese and Western Medicine, Shanghai University of Traditional Chinese Medicine, Tongde Hospital of Zhejiang Province, Wangjing Hospital of CACMS, Xiyuan Hospital CACMS, Chongqing Cancer Hospital, Gansu Provincial Cancer Hospital, and Shanxi Provincial Cancer Hospital. A total of 148 patients will be admitted to the treatment group with Western medicine and the integrated treatment group with traditional Chinese and Western medicine. The study schema and the flow chart are presented in Fig. 1. Inclusion and exclusion criteria are presented in Table 1. Two investigators will be responsible for the follow-up of each participant at baseline and every 6 weeks ± 1 week, and the data collection schedule is presented in Table 2.

**Table 1:**
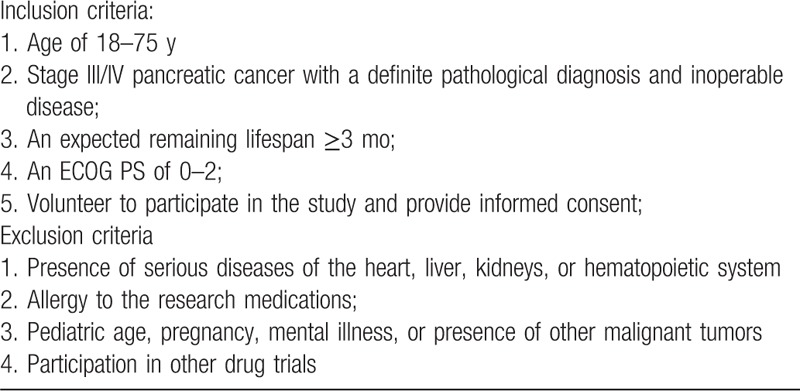
Patient inclusion and exclusion criteria.

**Table 2:**
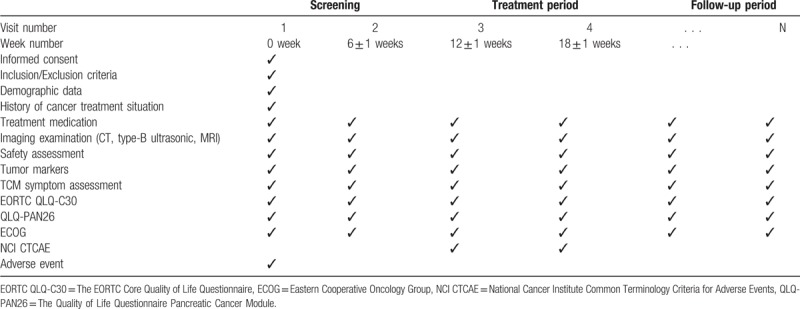
Data collection schedule.

### 3.2 Participants

All patients fulfilling the inclusion criteria will be recruited, and those who meet one or more of the exclusion criteria will be refused.

### 3.3 Shedding criteria

A researcher can decide to withdraw a patient from the study if:

1. The patient has an allergic reaction or serious adverse event.
2. During the study, the patient has other complications and special physiological changes, and it is not appropriate to continue in the study.
3. Subjects have poor compliance, or change their own drugs, or add non-specified combinations, especially those that have a greater impact on the study affecting the effectiveness and safety.

Subjects can quit on their own:

1. For whatever reason, the patient is unwilling or unable to continue the clinical research and makes a request to the doctor in charge to withdraw from the trial.
2. The subjects do not explicitly withdraw from the study, but they will not receive medication or follow-up.

Treatment of shed cases

For cases of withdrawal from the trial or loss to follow-up, the investigator should actively take measures to complete the last follow-up to analyze treatment efficacy and safety. For all cases of abscission, a case report form (CRF) and the cause of abscission should be completed.

### 3.4 Suspension of research standards

Study suspension means that the clinical research has not been completed according to the plan, and all studies are stopped midway. The purpose of the study suspension is mainly to protect the rights of the subjects, to ensure the quality of the research, and to avoid unnecessary economic losses. Criteria for discontinuing the study are as follows:

1. If serious safety problems occur in the study, the study should be suspended at that time.
2. Significant errors found in the clinical research protocol during the study make it difficult to evaluate drug effects, or there is an important deviation in the implementation of the research program and it is difficult to evaluate the drug effect after it continues.
3. Beijing municipal Science and Technology Commission suspends the research for some reason.

### 3.5 Sample size calculation

The sample size calculation formula is as follows: 


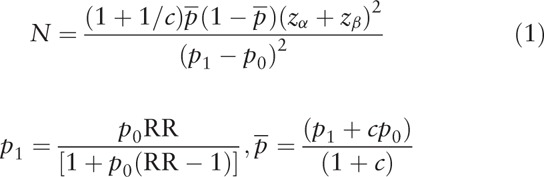


In the above formula, *N* is the number of samples in each group, *p*_0_ is the incidence of outcomes in the non-exposed group, *p*_1_ is the incidence of outcomes in the exposed group, *c* is the ratio of the number of non-exposed and exposed groups, *α* is an acceptable type I error, *β* is an acceptable type II error, and *Z*_α_ and Z_*β*_ can be determined by looking them up in the *Z* value table.

The 1-year survival rate is expected to be 22% in the non-exposed group and 45% in the exposed group. Assuming a non-exposed group and an exposed group sample ratio of 1:1, the required sample size is estimated by SAS 9.4 software (100 SAS Campus Drive Cary, NC) to be at least 134 cases in the 2 groups, based on a 2-sided alpha at a significance level of 0.05 and the test efficacy 1-beta of 80%. Meanwhile, considering a dropout rate of 15%, we concluded that a total of 148 patients would need to be recruited to ensure significant results.

### 3.6 Diagnosis and treatment

Diagnostic and staging criteria

1. Staging criteria: Refer to the American Joint Committee on Cancer (AJCC) staging classification for pancreatic cancer (2010, 7th edition).
2. Pancreatic cancer: There is a confirmed pathological diagnosis.
3. TCM syndrome diagnosis and efficacy evaluation: Refer to the “Guidelines for Clinical Research of New Drugs of Traditional Chinese Medicine (2002)” published by China Medical Science and Technology Press and “TCM Oncology (2nd Edition)” published by China Traditional Chinese Medicine Press.

Treatment plan

Western medicine treatment: refer to the standard treatment in the NCCN Guidelines 2017: pancreatic adenocarcinoma

Chinese medicine treatment: TCM syndrome differentiation.

### 3.7 Determination of grouping

Patients will be divided into a non-exposed group (Western medicine treatment group) and an exposed group (integrated traditional Chinese and Western medicine treatment group), according to whether they will receive TCM treatment or not. The classification of the cohort is based on the time of exposure after all clinical data are collected, not set at the time of enrollment.

1. Western medicine treatment group: times of chemotherapy ≥2 courses.
2. Integrated traditional Chinese and Western medicine treatment group: TCM treatment ≥12 weeks, times of chemotherapy ≥2 courses, TCM and Western medicine treatment can be performed simultaneously or before and after.

### 3.8 End point of observation

Patient death or the end of the study (October 31, 2021).

### 3.9 Division of treatment period and follow-up period

If the times of chemotherapy after enrollment are ≤4 courses, the follow-up period will be entered after the end of chemotherapy; if the times of chemotherapy after enrollment are >4 courses, the follow-up period will be entered after the fourth chemotherapy.

## 4 Study outcomes

### 4.1 Primary outcomes

#### 4.1.1 Overall survival (OS)

The primary outcome of this study is OS, which is from enrollment to patient death or the end of the study (October 31, 2021).

#### 4.1.2 Secondary outcomes

##### 4.1.2.1 Progression-free survival (PFS)

PFS, which is from enrollment to disease progression.

##### 4.1.2.2 Tumor markers

Tumor markers, including CA199, CEA, and AFP, are closely related to the treatment of pancreatic cancer. They are used as one of the indicators to evaluate the effectiveness of treatment and are recorded at baseline and at each treatment visit and follow-up periods.

##### 4.1.2.3 TCM symptom assessment

TCM symptom grading scale for pancreatic cancer is based on the common symptoms of pancreatic cancer. It is used to observe pre- and post-treatment symptom improvement and includes 13 symptoms: poor appetite, low food intake, fatigue, jaundice, nausea and vomiting, abdominal distension, pain, fever, spontaneous sweating, loose stools, constipation, and insomnia. Each symptom has 4 grades, namely: none, mild, moderate, and severe, with corresponding scores of 0, 1, 2, and 3. The higher the score, the more severe the symptom.

#### 4.1.3 Quality of life assessment

The EORTC Core Quality of Life Questionnaire (EORTC QLQ-C30) is a core scale for quality of life analysis of cancer patients developed by The European Organization for Research and Treatment of Cancer (EORTC). Because of its reliability and validity, it is widely used in the assessment of quality of life of all cancer patients. The Quality of Life Questionnaire pancreatic cancer module (QLQ-PAN26) is a module designed according to specific characteristics of pancreatic cancer and is used to evaluate the symptoms, side effects, and quality of life of pancreatic cancer patients. It has been widely used in clinical trials and studies of pancreatic cancer in recent years.^[15,16]^

EORTC QLQ-C30 consists of 5 functional subscales (body, role, cognitive, emotional, and social functions), 3 symptom subscales (fatigue, pain, and nausea and vomiting), and a general health status subscale, as well as several single items. The higher the scores of functional subscales and general health status scale, the better the quality of life. The higher the score of symptom subscale, the worse the quality of life. QLQ-PAN26 includes cancer pain, digestive symptoms, and satisfaction with health care. The higher the severity of these indicators, such as fatigue and pain, the higher the score, suggesting poorer quality of life for pancreatic cancer patients.^[17]^

#### 4.1.4 Body condition assessment

Eastern Cooperative Oncology Group (ECOG) in the United States has developed a simplified activity status rating scale, which is an indicator for evaluating the general health status and treatment tolerance of patients with grades 0 to 5. It is generally considered that patients with activity level 3 or 4 are not suitable for chemotherapy.^[18]^

#### 4.1.5 Imaging assessment

Imaging evaluation includes the application of CT, type-b ultrasonic, MRI, etc to objectively evaluate the therapeutic effect.

Each participant has a CRF, and all relevant data will be recorded in it. Two clinical research associates supervise the whole research process.

### 4.2 Safety assessment

Routine blood tests and liver and kidney function tests are assessed at baseline, and functional damage is assessed at each follow-up visit. In addition, the investigator will ask subjects at each visit whether there are any adverse events (AEs), including physical discomfort or impairment. If there are any AEs, the investigator would provide appropriate treatment to the patient immediately and record it in the CRF at that time. Adverse effects are evaluated according to the National Cancer Institute (NCI) Common Terminology Criteria for Adverse Events.

### 4.3 Data management

#### 4.3.1 Data recording

The data are required to be filled in uniform CRFs completely and accurately in a timely manner without missing items.

#### 4.3.2 Data monitoring

During the study, the supervisor will: check the informed consent and screening and inclusion status of the subjects in the cooperating units regularly, confirm that the filled content of the CRF is consistent with the original data, verify the withdrawal and loss of the enrolled subjects, record the reasons in the CRF, and confirm that all adverse events are documented.

#### 4.3.3 Data management

Data managers are responsible for data entry and data re-verification. If problems are found, they should be registered and reported in time so as to deal with them quickly. All errors and modification of results should be recorded and properly preserved to ensure the quality of the data.

#### 4.3.4 Data analysis

All statistical analyses will be performed using Statistical Packages of Social Sciences software (SPSS). A one-sided test (validity) will be used for all major efficacy indicators, and *P* values ≤.05 will be considered statistically significant. The measurement data will be expressed as the mean ± standard deviation, and the count data will be expressed using frequency and percentage. For quantitative indicators such as age, it is necessary to check whether the data meet the precondition of normality and homogeneity of variance. If the above conditions are met, a *t* test of measurement data of group design will be adopted. If only normality and not homogeneity of variance are satisfied, the calibration *t* test of measurement data in group design will be adopted. Otherwise, the Wilcoxon rank sum test will be used. For qualitative indicators such as sex, the general chi-square test will be performed according to the statistical analysis method corresponding to the data of the contingency table. If there is a frequency with a theoretical frequency <5, Fisher exact test will be used to directly calculate the exact probability. For survival data, the survival limit will be used to calculate the survival times of 25%, 50%, and 75% of each cohort. The log-rank test will be used to compare the survival rate, and the corresponding survival curve will be obtained.

For the analysis of influencing factors of efficacy, the univariate Cox regression model will be adopted in the first screen (*P* < .10), and the screened variables will then be fitted to the multivariate Cox regression model (*P* < .05).

#### 4.3.5 Patient and public involvement

Patients and the general public were not involved in the design of the present study. Patients will be involved in the data collection process by providing information during data collection.

#### 4.3.6 Ethics and dissemination

This work was supported by Beijing Municipal Science and Technology Commission and approved by the ethics committee of Guang’anmen Hospital, China Academy of Chinese Medical Sciences (Approval No. 2019-016-KY). All patients will sign a written informed consent prior to data collection. The results will be disseminated through peer-reviewed journals and conference presentations and will be openly shared after completion of the trial.

## 5 Discussion

TCM has been used in China for thousands of years. In recent years, it has been gradually accepted by Western countries.^[19]^ In the literature,^[20,21]^ it has been reported that TCM plays an important role in reducing the side effects of chemotherapy and improving the survival of patients. In addition, studies^[22]^ have reported that TCM combined with chemotherapy can improve survival rates and quality of life. This study aims to assess the survival and quality of life of patients with advanced pancreatic cancer, refine the core pathogenesis of TCM for pancreatic cancer and core prescriptions, and ultimately provide reliable data support for the formation and application of clinical solutions for the treatment of pancreatic cancer using integrated Chinese and Western medicine.

### 5.1 Strengths

This study has several advantages. First, this is a multicenter prospective observational cohort study involving 12 medical units in 9 provinces and cities in China, all of which are grade-3A hospitals. The results are reliable and can provide relatively objective evidence. Second, observational studies are more humane than randomized controlled trials (RCT). Moreover, it will lay a foundation for the standardization of integrated Chinese and Western medicine treatment to improve the curative effect, reduce the medical expenses of pancreatic cancer and the abuse and reuse of drugs, and generate certain social benefits for more patients with pancreatic cancer. This study involving 12 medical units in 9 provinces and cities in China, all of which are grade-3A hospitals, will generate relatively reliable results.

### 5.2 Limitations

This study also has some limitations. The present study is a prospective cohort study with a small patient sample size. However, because of the lower incidence of pancreatic cancer relative to other cancers, the number of patients with advanced pancreatic cancer meeting the inclusion and exclusion criteria may be relatively small, which will result in a small sample size. Given the small sample size, the amount of data that can be collected from the integrated Chinese and Western medicine group may lead to results that are not as robust as would be feasible with a larger sample size. Because of the short study duration and funding constraints, only 148 advanced pancreatic cancer patients were eventually recruited. However, even after the end of the study, we will continue to follow the survival of subjects so as to more accurately analyze and evaluate the clinical efficacy of integrated Chinese and Western medicine in the treatment of pancreatic cancer.

## Acknowledgments

The authors thank all the research centers and their staff for their hard work in making this research possible.

## Author contributions

Juling Jiang wrote the first draft of the article of the study protocol. Rui Liu, Qiujun Guo, and Baojin Hua are responsible for writing, review, and editing. Zhenhua Zhang, Xiwen Zhang, Runzhi Qi, Shuntai Chen, Honggang Zheng, and Baojin Hua are responsible for managing the project and conducting a formal analysis. Xing Zhang and Yupeng Xi are responsible for data curation. Baojin Hua received the funding to ensure that this study could go forward. All authors have contributed to the design and implementation of the study.
